# Designed-seamless irradiation technique for extended whole mediastinal proton-beam irradiation for esophageal cancer

**DOI:** 10.1186/1748-717X-7-173

**Published:** 2012-10-19

**Authors:** Noriyuki Okonogi, Takatuki Hashimoto, Masaya Ishida, Toshiki Ohno, Toshiyuki Terunuma, Toshiyuki Okumura, Takeji Sakae, Hideyuki Sakurai

**Affiliations:** 1 Proton Medical Research Center, and Department of Radiation Oncology, University of Tsukuba, 1-1-1 Tennodai, Tsukuba, Ibaraki, 305-8575, Japan; 2 Department of Radiation Oncology, Gunma University Graduate School of Medicine, 3-39-22 Showa-machi, Maebashi, Gunma, 371-8511, Japan

**Keywords:** Proton-beam therapy, Esophageal cancer, Matching field

## Abstract

**Background:**

Proton-beam therapy (PBT) provides therapeutic advantages over conformal x-ray therapy in sparing organs at risk when treating esophageal cancer because of the fundamental physical dose distribution of the proton-beam. However, cases with extended esophageal lesions are difficult to treat with conventional PBT with a single isocentric field, as the length of the planning target volume (PTV) is longer than the available PBT field size in many facilities. In this study, the feasibility of a practical technique to effectively match PBT fields for esophageal cancer with a larger regional field beyond the available PBT field size was investigated.

**Methods:**

Twenty esophageal cancer patients with a larger regional field than the available PBT single-field size (15 cm in our facility) were analyzed. The PTV was divided into two sections to be covered by a single PBT field. Subsequently, each PTV isocenter was aligned in a cranial-caudal (CC) axis to rule out any influence by the movement of the treatment couch in anterior-posterior and left-right directions. To obtain the appropriate dose distributions, a designed-seamless irradiation technique (D-SLIT) was proposed. This technique requires the following two adjustments: (A) blocking a part of the PTV by multi-leaf collimator(s) (MLCs); and (B) fine-tuning the isocenter distance by the half-width of the MLC leaf (2.5 mm in our facility). After these steps, the inferior border of the cranial field was designed to match the superior border of the caudal field. Dose distributions along the CC axis around the field junction were evaluated by the treatment-planning system. Dose profiles were validated with imaging plates in all cases.

**Results:**

The average and standard deviation of minimum dose, maximum dose, and dose range between maximum and minimum doses around the field junction by the treatment-planning system were 95.9 ± 3.2%, 105.3 ± 4.1%, and 9.4 ± 5.2%. The dose profile validated by the imaging plate correlated with the results of the treatment-planning system in each case, with an error range within 4.3%.

**Conclusions:**

Dose distributions around the field junction were applied using D-SLIT. D-SLIT can be a useful treatment strategy for PBT of extended esophageal cancer.

## Background

Proton-beam therapy (PBT) appears to provide distinct therapeutic advantages over conformal x-ray therapy in sparing organs at risk (OAR) when treating esophageal cancer [1,2]. These advantages are based on the fundamental physical dose distribution of the proton-beam [3]. However, cases with extended esophageal lesions or with distant regional lymph node metastases are hard to treat with conventional PBT with a single isocentric field. This is mainly due to the fact that the length of the planning target volume (PTV) is longer than the available field size of PBT in many facilities.

On the other hand, some studies have reported so-called ‘patch-field’ strategies for tumors with highly complex shapes (e.g., a target coverage wrapped around a critical structure) in PBT [4,5]. These strategies were used to confirm dose delivery to the target and spare OAR. In principle, target regions are divided into segments, each treated by a separate proton field. Subsequently, the distal edge of one field is matched with the lateral field edge of the second field. However, few studies have reported a long field for such extended esophageal cancer.

The present study investigated the feasibility of a practical technique to effectively match PBT fields along the lateral edge for esophageal cancer with larger regional fields beyond the available PBT field size.

## Methods

### Therapy beams and systems

The PBT system used consists of an isocentric rotational gantry equipped with an x-ray imager, a rotational treatment couch, a treatment-planning system (HITACHI 3D Treatment-planning system version 1.72, Tokyo, Japan), a treatment-planning computed tomography (CT) scanner, and an x-ray simulator without any system modification [6]. System precision studies were carried out in order to ensure that there was no deviation of the isocenter while moving the treatment couch loaded with a 60-kg phantom.

Treatment was delivered via 200 MeV proton beams during the end-expiratory phase using a respiratory gating system (Anzai Medical Co., Tokyo, Japan) [7]. The patient’s body was immobilized using an individually shaped body cast (ESFORM; Engineering System Co., Matsumoto, Japan). Respiratory gating was controlled by laser range finder monitoring the movement of the patient’s body surface.

### Treatment-planning methods of D-SLIT

The present study proposes the novel ‘Designed-Seamless Irradiation Technique (D-SLIT)’ for longitudinally extended PTV in PBT. The procedures for this technique are described below.

#### Acquiring CT images at 5-mm intervals during the expiratory phase under a respiratory gating system

All images were used to design treatment plans by a single treatment-planning system.

#### After delineation the PTV was divided into two sections covered by a single PBT field (cranial PTV and caudal PTV (Figure 1))

**Figure 1:**
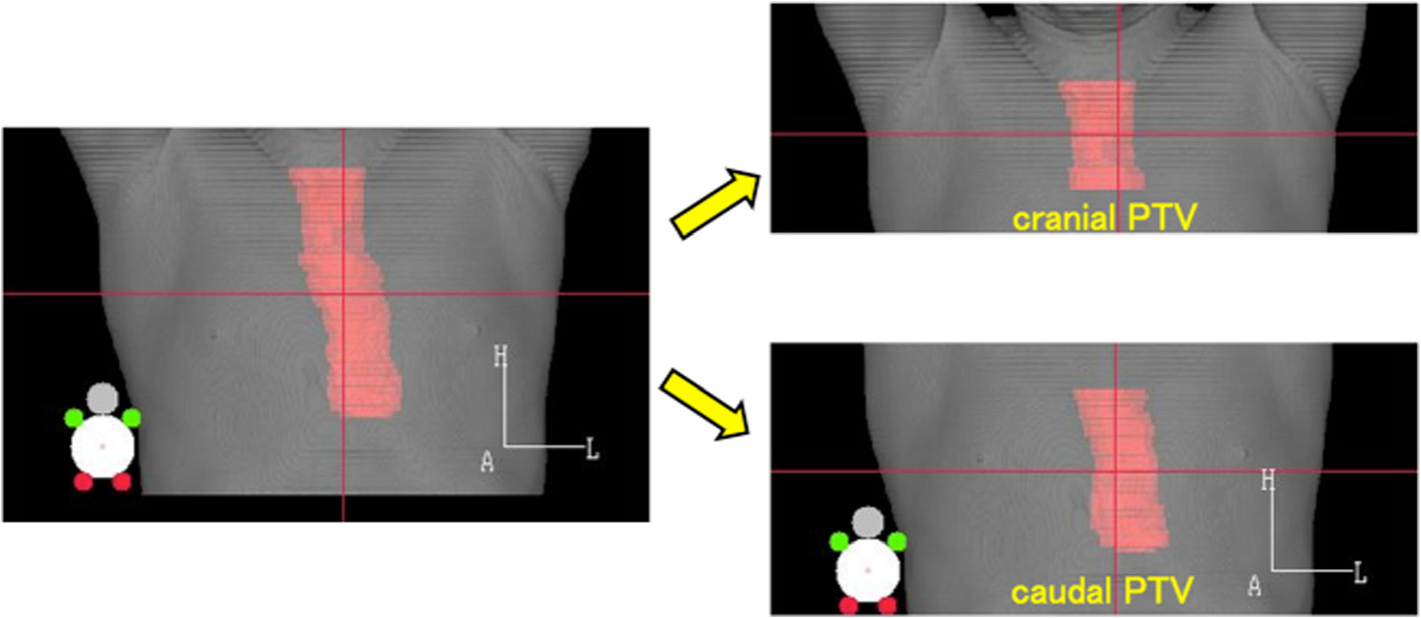
**Definition of PTV.** CTV included the primary tumor plus a 30-mm cranial-caudal margin, and included the metastatic lymph nodes plus a 10-mm margin. PTV encompassed the CTV with a 5- to 10-mm margin in all directions. PTV was divided into two sections covered by a single PBT field: cranial PTV and caudal PTV.

In this study, target volumes were defined and delineated in a self-consistent manner in order to reduce biases. Gross tumor volume was defined as the volume of a primary tumor demonstrated by CT and endoscopy, as well as metastatic lymph nodes that measured ≥10 mm in the long axis. The clinical target volume (CTV) included the primary tumor plus a 30-mm cranial-caudal margin, and included the metastatic lymph nodes plus a 10-mm margin [8]. PTV encompassed the CTV with a 5- to 10-mm margin in all directions (10-mm margin in the cranial-caudal direction).

#### Each PTV isocenter was aligned along the CC axis

Two PTV isocenters were aligned on the cranial-caudal (CC) axis in order to avoid any influence by the movement of the treatment couch in the anterior-posterior and left-right directions. In this study, each plan included two co-planar, equally weighted beams placed at gantry angles of 0° and 180° for each PTV. An additional 10-mm margin was included to cover each PTV by enlarging the multi-leaf collimator (MLC) and adjusting the range shifter. The snout is close to the patient in the treatment-planning system and hence reduces lateral penumbra of proton beams, in principle.

#### Blocking part of the PTV by movement of the MLC and fine-tuning the isocenter distance

**Figure 2:**
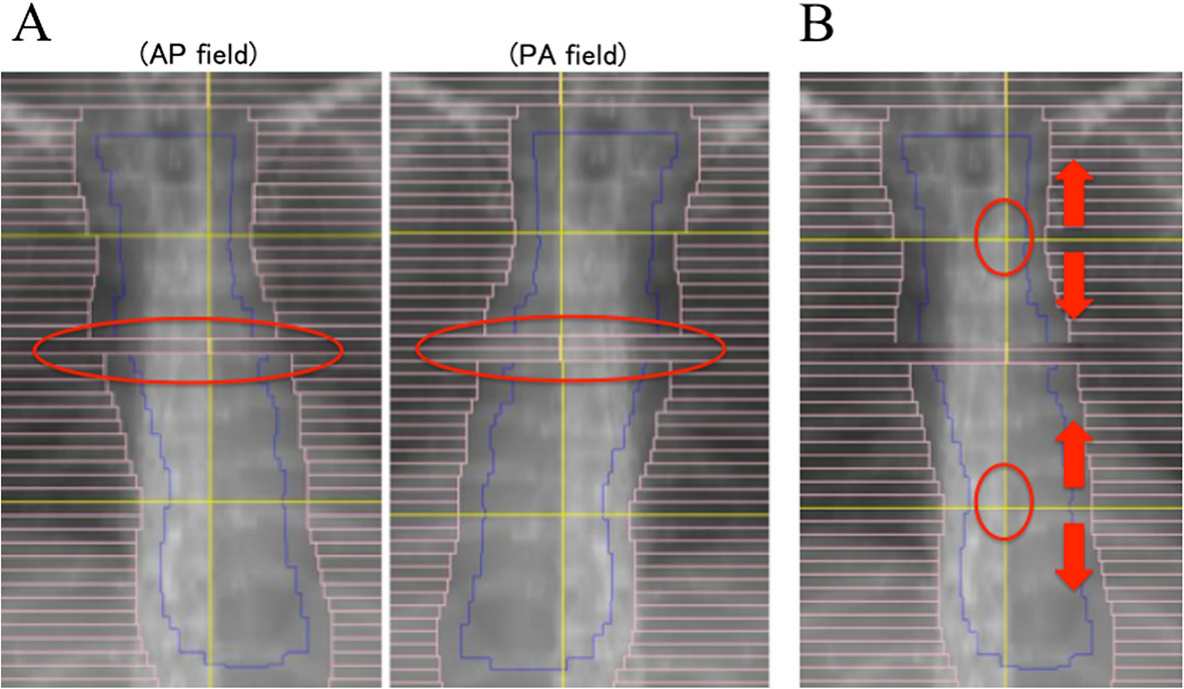
**D-SLIT fine-tuning.** The two figures show beam’s eye view-shaped fields designed to match the cranial field and caudal field. (**A**) To avoid hot spots around the junction, a part of the PTV was blocked by the MLC. (**B**) To reduce hot or cold spots, the isocenter distance was fine-tuned by 2.5 mm (half-width of the MLC leaf in our facility).

Proton beams have a few inherent lateral penumbras. Therefore, the risk of hot or cold spots around the field junction cannot be avoided. To minimize such hot and cold spots, fine-tuning is required (Figure 2). In order to obtain acceptable dose distribution, at first, one or two MLC leaves were moved to block part of the PTV at the junction in both the anterior-posterior (AP) and posterior-anterior (PA) directions. After checking dose distribution in several patterns, the most acceptable pattern (e.g., 5-mm blocking in the AP field and 10-mm blocking in the PA field) was selected (Figure 2A). Secondly, the isocenter distance was fine-tuned by the half-width of the MLC leaf (2.5 mm in our facility) for further homogeneities at the field junction (Figure 2B).

### Patient selection

This study was conducted in accordance with the ethical standards defined in the Declaration of Helsinki. PBT for esophageal cancer was approved by the ethics committee of the University of Tsukuba. A total of 20 cases from our institutional records were analyzed in this study. All patients had middle thoracic esophageal cancer, and gastro-esophageal junction cancers were not included. All patients provided written informed consent after a comprehensive discussion covering the nature of their illness, other therapeutic options, and potential adverse effects. Eight of the 20 cases were finally irradiated with D-SLIT. The remaining 12 cases only underwent simulated D-SLIT based on previously acquired CT.

### Actual treatment of eight patients with on-going planning

The photon equivalent dose (Gray equivalent dose; GyE) was defined as the physical dose (Gy) × the relative biological effectiveness of the proton beam. Based on the biological response of salivary gland tumor cells, the relative biological effectiveness of the proton beam was assigned a value of 1.1. The planned total doses were 60.0 to 70.0 GyE (median, 60.0 GyE), at 2.0 GyE per fraction. This was the common radiation dose and fractionation schedule for esophageal cancer patients treated with PBT in our institute. Field junctions were shifted twice along the CC direction.

### Evaluation methods

**Figure 3:**
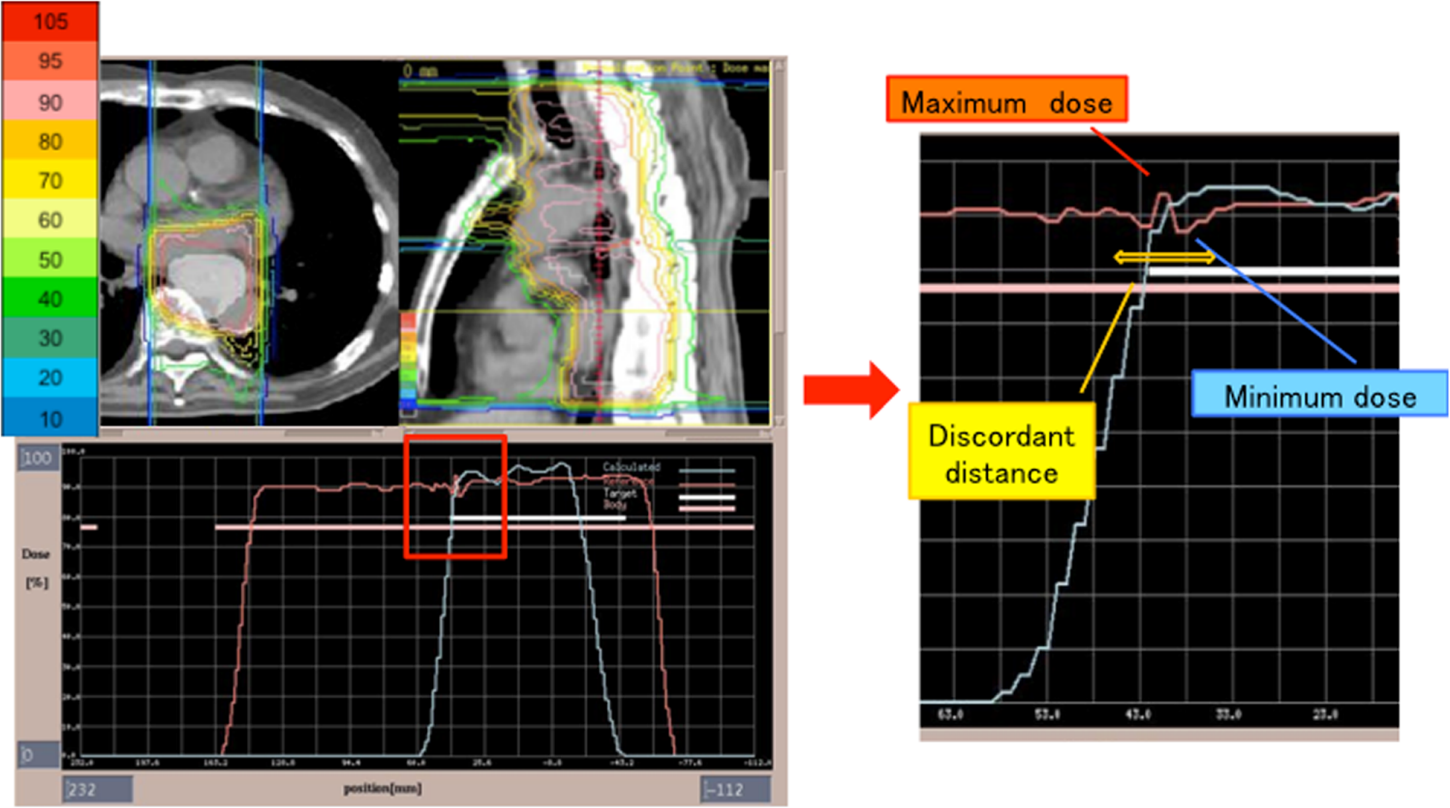
**D-SLIT dose distribution and profile.** (Left) The upper figure shows dose distribution with D-SLIT in the axial view and sagittal view. PTV appears white in the axial view. Dose distributions in percentages (10-105%) are shown in the left bar chart. The lower figure shows the dose profile along the CC axis on the isocenter line. (Right) Enlarged dose profile around the field junction. Minimum dose, maximum dose, and discordant distance were calculated by treatment-planning system. To calculate discordant distance, all dose profiles were exported to a spreadsheet. At first, the dose increasing/decreasing region over 2% was extracted at intervals of 3-mm on the dose profile around field junction. Secondly, the starting points of does change in those regions were detected with 0.1-mm resolution. Then, the distance between two furthest points was calculated as the discordant distance.

For evaluation of the feasibility of this technique, the dose distributions and profiles along the CC axis around the field junction were compared in the treatment-planning system. Regarding dose profiles, the average and standard deviation (S.D.) of minimum dose, maximum dose, and dose range between the maximum and minimum dose were evaluated. Additionally, the inhomogeneous, non-uniform dose distribution range around the field junction, termed the ‘discordant distance’, was evaluated (Figure 3). Imaging plates (IP) consisting of storage films coated with photostimulated phosphor were used for validation of dose profiles. Adverse events of the eight patients receiving PBT were evaluated by an outpatient clinician using Common Terminology Criteria for Adverse Events (CTCAE) version 4.0.

## Results

**Figure 4:**
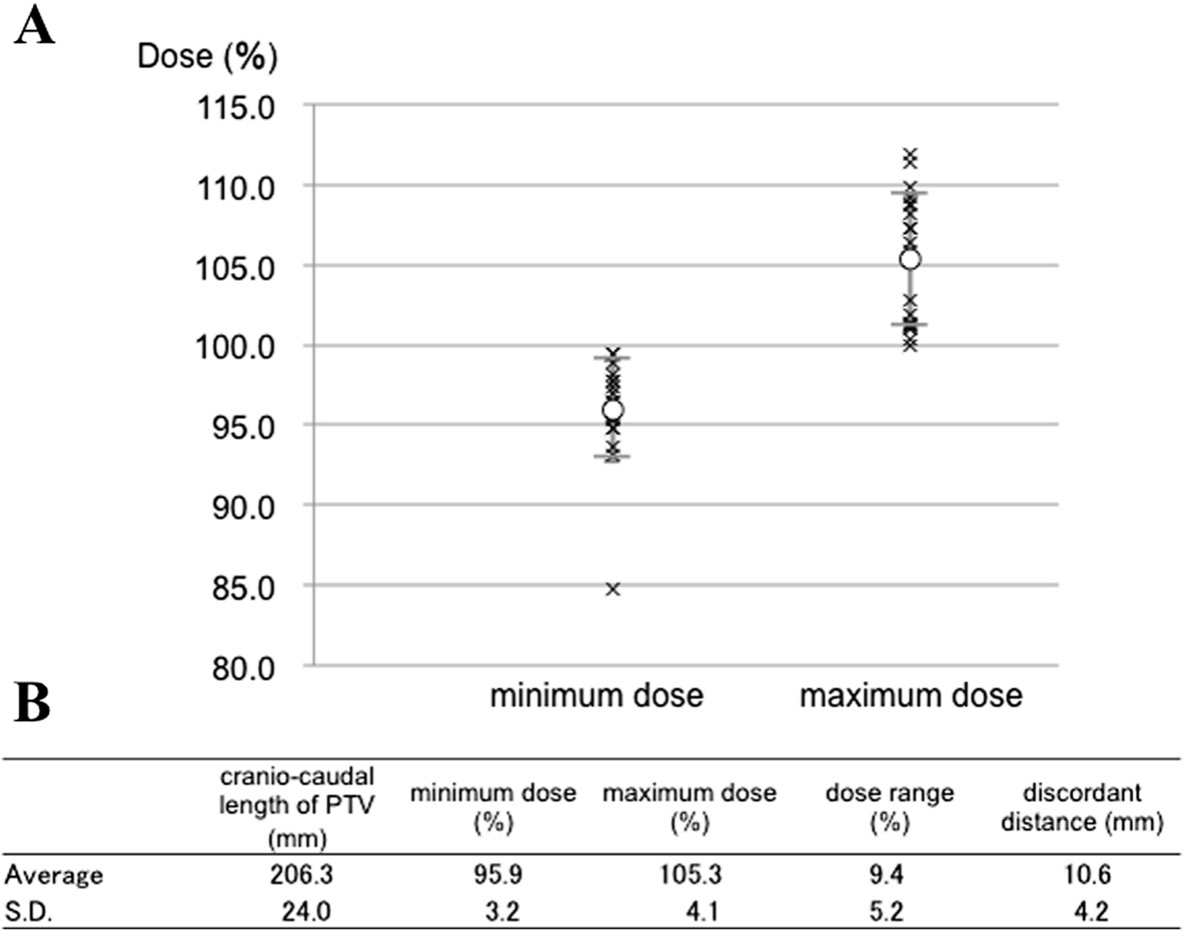
**Results of average and standard deviation with D-SLIT.** (**A**) Distributions of minimum dose and maximum dose in all 20 cases. Each cross symbol shows the dose in each case. Open symbols represent average doses. (**B**) Average and standard deviation with D-SLIT.

**Figure 5:**
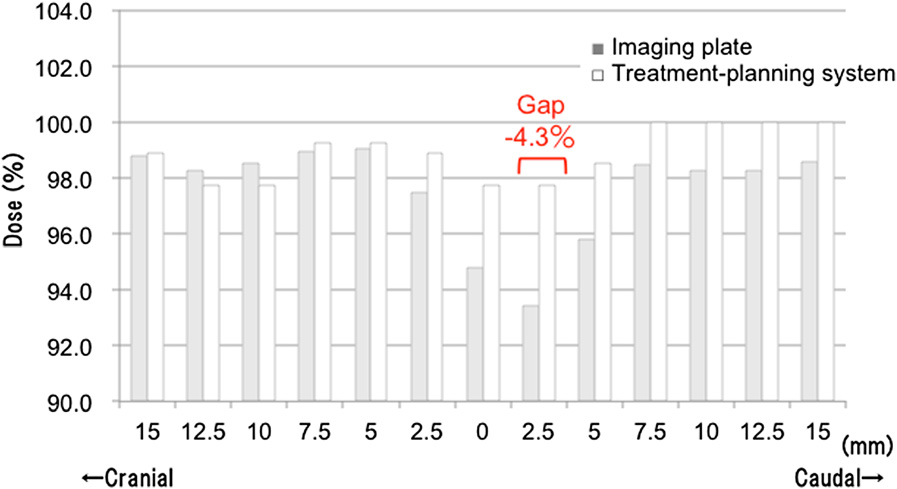
**Comparison of dose distributions between the treatment-planning system and imaging plate.** Dose distribution by the treatment-planning system and imaging plate (IP) along the CC axis on the isocenter line. White bars show the treatment-planning system, and gray bars show the imaging plate. The dose ‘gap’ between the treatment-planning system and IP was up to 4.3%.

All 20 cases were planned with D-SLIT. It usually took an extra 2–3 hours to produce a D-SLIT plan in each case. Target volumes were covered well in all plans. Physical characteristics of the proton beam resulted in a lower dose to a particular OAR, such as the lung, heart or spinal cord. A representative case of dose distribution by the PBT plan with D-SLIT is presented in Figure 3. Although a slightly hot region was seen in front of the vertebral body, the dose distribution around the field junction was almost homogeneous. On the other hand, the spinal cord received less than 60% of the prescribed dose and a small volume of the lung received approximately 20% of the prescribed dose. Figure 4A shows a distribution chart of the minimum and maximum doses, and Figure 4B shows the average ± S.D. of PTV length, minimum dose, maximum dose, dose range, and discordant distance around the field junction along the CC axis on the isocenter line. Only one case was outside the minimum dose distribution chart, with 84.8% of the prescribed dose. The average and S.D. in minimum dose, maximum dose, and dose range were 95.9 ± 3.2%, 105.3 ± 4.1%, and 9.4 ± 5.2%, respectively. The average ± S.D. of the discordant distance was 10.6 ± 4.2 mm. Nine patients presented with Dmax >107% and five patients presented with Dmin <95% (Table 1). The dose profile validated by IP correlated with the result of the dose profile in the treatment-planning system for each case, and the error range was within 4.3% (Figure 5). The 20-80% penumbra width within the region of the spread out Bragg peak was approximately 7 to 8 mm using 200 MeV protons in this study. To avoid hot or cold spots around the junction, the field junctions were sifted by 15–20 mm twice during an entire treatment course in eight patients treated with D-SLIT. Acute toxicity could be evaluated in these patients, and grade ≥3 treatment-related toxicity was not observed around the field junction (unpublished data).

**Table 1:** Dosimetric results in 20 cases

**Case No.**	**Length of PTV (mm)**	**Minimum dose (%)**	**Maximum dose (%)**	**Discordant distance (mm)**	**Homogeneityindex of PTV**
1	240	95.6	107.3	18.1	1.12
2	195	95.3	101.9	11.3	1.07
3	230	95.4	101.0	9.5	1.06
4	190	97.7	108.2	13.3	1.11
5	200	96.4	101.1	2.9	1.05
6	190	99.5	108.7	12.9	1.09
7	170	99.5	106.3	14.8	1.07
8	190	97.8	100.0	5.0	1.02
9	210	95.9	101.4	8.1	1.06
10	220	94.8	108.9	14.3	1.15
11	165	97.0	109.2	12.5	1.13
12	215	93.7	100.3	9.3	1.07
13	230	94.8	102.8	10.9	1.08
14	230	84.8	109.8	15.9	1.29
15	245	98.2	111.9	12.8	1.14
16	190	96.5	101.2	3.0	1.05
17	210	98.9	111.4	13.0	1.13
18	165	95.7	105.9	11.0	1.11
19	220	97.4	101.2	5.0	1.04
20	220	93.1	107.3	8.8	1.15
Average	206.3	95.9	105.3	10.6	1.10
S.D.	24.0	3.2	4.1	4.2	0.1

Abbreviations: No.; number, PTV; planning target volume, S.D.; standard deviation.

## Discussion

Esophageal cancer is the eighth most common cancer in Japan [9]. Despite modern multimodality therapy, esophageal cancer still has a poor prognosis [10]. Surgical therapy remains the mainstay of curative therapy for esophageal cancer. However, many patients with esophageal cancer are diagnosed at an advanced, inoperable stage. Furthermore, some patients may be deemed unsuitable for surgery because of co-morbidity or increasing age [11]. On the other hand, some studies have reported that definitive chemoradiotherapy (CRT) is an effective alternative to surgical therapy and can achieve long-term disease control, with the overall 5-year survival rate reaching 20-27% for locally advanced esophageal cancer [12,13].

Conventional CRT for advanced esophageal cancer is generally challenging because of the surrounding radiosensitive organs such as the lung, and the proximity of critical structures such as the heart and spinal cord. In order to reduce the risk of morbidity in these organs, PBT has advantages based on the fundamental physical dose distribution [3]. As described above, however, the available field size of PBT is insufficient to treat locally advanced esophageal cancer existing as an extended esophageal lesion. Field sizes up to 25 × 25 cm^2^ can be achieved with the double scattering system [14]. This problem is common to many facilities.

On the other hand, the so-called ‘patch-field’ technique has been introduced in several studies [4,5,15,16]. This technique is used to optimize dose distribution within an irregular volume in close proximity to critical normal structures. Hug et al. reported excellent sparing of the lens and selected intraorbital and ocular normal structures, while maintaining conformal dose-target coverage in orbital rhabdomyosarcoma of children [4]. According to their report, the target volume was divided into two segments, each treated by a separate radiation field. Utilizing sharp dose distribution, the distal edge of one field was matched with the lateral field edge of the second field while taking care to avoid match lines in critical structures. In fact, this method provided long disease-free periods, without severe morbidity.

In the present study, the distal or cranial field edge of one field was matched with that of the other field. To date, several studies concerning PBT for esophageal cancer have been reported, and to our knowledge, this study is the first report on PBT using ‘patch-field’ technique for esophageal cancer. Traditionally, distal-cranial matching has been used for craniospinal irradiation with photons as well as other diseases [17,18]. However, hot or cold spots around the field junction cannot be avoided. On the other hand, in the present study, the average minimum dose and maximum dose was between 95.9% and 105.3% around the field junction along the CC axis by the treatment-planning system. This result is acceptable compared with the dose homogeneities of previous reports using the ‘patch-field’ technique [4,5], and satisfies the criteria of the International Commission on Radiation Units reports 50 and 62 [19,20].

In some cases in the present report, hot or cold spots were actually present in the target volume around the field junction. Passing through highly complex heterogeneities and some minor inherent lateral penumbra of protons could have caused the dose distribution at the field junction to be non-uniform. Therefore, shifting the field junction should be considered to avoid over- or under-dosage. In the present study, the average ± S.D. of discordant distance was 10.6 ± 4.2 mm. From these results, it seems necessary to slide the field junction by at least 19.0 mm (two-sided 95% confidence interval).

The present study has several limitations. In particular, the effects of respiratory and peristaltic motions on the esophagus were not well considered. Yaremko et al. reported esophageal motion using respiratory-gated four-dimensional CT [21]. In their study, the mean of peak-to-peak displacement of the esophagus was 7.1 mm in the CC axis in 31 consecutive patients treated for esophageal cancer. Although the present report used CT images obtained during the expiratory phase under a respiratory gating system, it is insufficient to evaluate motion of the esophagus within the expiratory phase and to evaluate peristaltic motion during treatment. Shifting the field junction several times is one possible way to avoid the uncertainty due to these motions in clinical practice. Further physical and clinical studies are needed.

## Conclusions

Dose distributions around the field junction were determined using D-SLIT. D-SLIT might represent a novel and safe therapeutic option for locally advanced esophageal cancer. Although further physical and clinical studies are needed to confirm the effects of D-SLIT, this approach might be a useful treatment strategy for PBT of extended esophageal cancer.

## Abbreviations

PBT: Proton-beam therapy; PTV: Planning target volume; CC: Cranial-caudal; D-SLIT: Designed-seamless irradiation technique; MLC: Multi-leaf collimator; OAR: Organs at risk; CT: Computed tomography; CTV: Clinical target volume; AP: Anterior-posterior; PA: Posterior-anterior; IP: Imaging plate; CRT: Chemoradiotherapy.

## Competing interests

The authors declare that they have no competing interests.

## Authors' contributions

TH and HS coordinated the entire study. Patient clinical data collection was done by NO and TO (4th author). Treatment planning was conducted by NO, TH and TO (4th author). Data collection of dose profile was worked out by MI and TT. Data analysis was done by NO, MI and TS. The manuscript was prepared by NO. Corrections and/or improvements were suggested by TH, TO (6th author) and TS. Major revisions were done by HS. All authors read and approved the final manuscript.
